# A pilot study comparing superficial wound swab, deep tissue biopsy and fine needle aspiration biopsy in identifying infecting organisms in foot ulcers due to diabetes

**DOI:** 10.1186/1757-1146-4-S1-P4

**Published:** 2011-05-20

**Authors:** Yusuf Bhabha, Paul Tinley, Peter Davoren, Petra Derrington

**Affiliations:** 1 Gold Coast Hospital, Southport, Australia; 2 Charles Sturt University, Albury, Australia

## Background

Current clinical practice widely regards deep tissue biopsy as the gold standard for identification of wound bacterial bio-burden. This study aims to establish whether fine needle aspiration biopsy (FNAB) is as accurate as deep tissue biopsy and therefore offers a more accurate, cheaper and suitable alternative to routinely used superficial swab in diabetic wounds of varying depth and severity.

## Methods

A total of 15 infected diabetic foot wounds were sampled and cultured. Three specimens were taken from each wound: superficial swab before debridement, followed by fine needle aspiration biopsy and deep tissue specimen at the end of sharp debridement.

## Results

FNAB identified the same significant bacteria found in deep tissue culture in only 5 (33%) wounds missing all or some significant bacterial isolates in 10 (67%) wounds. In 6 (40%) of these wounds FNAB cultures were negative. In comparison swab cultures identified the same significant bacterial isolates found in deep tissue culture in 13 (87%) wounds, missing 1 bacterial isolate in 2 (13%) wounds with no negative cultures recorded.

## Conclusions

In this limited sample it would appear that FNAB culture technique is severely inadequate in identifying pathogens in diabetes foot wounds in comparison to superficial swab technique and gold standard deep tissue biopsy. FNAB cultures missed microorganisms in two thirds of wounds and had a high false negative rate.

